# SAP—A Sequence Mapping and Analyzing Program for Long Sequence Reads Alignment and Accurate Variants Discovery

**DOI:** 10.1371/journal.pone.0042887

**Published:** 2012-08-07

**Authors:** Zheng Sun, Weidong Tian

**Affiliations:** State Key Laboratory of Genetic Engineering, Institute of Biostatistics, School of Life Sciences, Fudan University, Shanghai, China; Indiana University, United States of America

## Abstract

The third-generation of sequencing technologies produces sequence reads of 1000 bp or more that may contain high polymorphism information. However, most currently available sequence analysis tools are developed specifically for analyzing short sequence reads. While the traditional Smith-Waterman (SW) algorithm can be used to map long sequence reads, its naive implementation is computationally infeasible. We have developed a new Sequence mapping and Analyzing Program (SAP) that implements a modified version of SW to speed up the alignment process. In benchmarks with simulated and real exon sequencing data and a real *E. coli* genome sequence data generated by the third-generation sequencing technologies, SAP outperforms currently available tools for mapping short and long sequence reads in both speed and proportion of captured reads. In addition, it achieves high accuracy in detecting SNPs and InDels in the simulated data. SAP is available at https://github.com/davidsun/SAP.

## Introduction

Next-generation sequencing (NGS) technologies, such as the second-generation NGS including Roche 454, Illumina (Solexa) and ABI SOLiD, and the third-generation NGS including Pacific Bioscience' single molecular sequencing, are transforming today's biology [1]. To fully take advantage of NGS to accelerate today's biological research, a key challenge is to develop efficient and accurate algorithms that can handle and analyze large-scale sequence data produced by NGS. Currently, the widely used algorithms for mapping sequence reads onto a reference genomes can be generally classified into two categories, those using a hash table for indexing the reference genome, such as BLAT [2], MAQ [3], SHRiMP [4] and ZOOM [5], and those implementing Burrows Wheeler transform (BWT)-based techniques for indexing [6], such as BWA [7], BOWTIE [8] and SOAP2 [9]. BWT-based methods create an efficient index structure of the reference genome, making them running much faster with low memory consumption than hash table-based methods. For example, BOWTIE reported a 30-fold increase in computational speed, compared to MAQ [8]. However, these algorithms often allow only a limited number of mismatches in order to speed up the mapping process of short sequence reads, making them inappropriate for identifying genomic regions with high polymorphism rates. On the other hand, they are exclusively designed for aligning reads no longer than 100 bp, limiting their usefulness to meet the needs of mapping long reads generated by the newest NGS technologies.

The Smith-Waterman (SW) algorithm calculates every possible combination of alignment, and guarantees to find the best alignment [10]. However, SW is time-consuming with a complexity of *O(nm)* where *n* and *m* are the length of sequence read and reference sequence, respectively, making it computationally inefficient for mapping huge number of sequence reads against the reference genome. SHRiMP, a short read mapping algorithm, introduced a simplified Smith-Waterman algorithm by “seeding” a sequence read to a given genomic region and then determining the exact placement of the sequence reads [4]. However, in our experiment SHRiMP is found to be very time-consuming. Based on the BWT transformation, Lam et al. [11] developed the BWT-SW algorithm that runs dynamic programming to align two FM-indices [12] created from BWT-transformed suffix tree, and is equivalent to SW yet thousands of times faster. Li and Durbin [13] furthered the BWT-SW idea, and developed BWA-SW for aligning long sequence reads. BWA-SW introduces heuristics during the seeding process by only allowing extension of alignment for high-scoring seeds, and was found to be more accurate and faster than Blat for mapping long sequence reads [2].

In this study, we have developed a new alignment tool named SAP (**S**equencing mapping and **A**lignment **P**rogram). SAP contains two component algorithms: the SAP Mapper and the SAP Predictor for mapping sequence reads and predicting polymorphisms, respectively. In SAP Mapper, a hash table is used to index the sequence reads. Then, when aligning the sequence reads, different from other algorithms, a modified SW is used by taking advantage of the hashing structure during the seeding process, which reduces its complexity to approximately O(n) and greatly speeds up the mapping process. The modified SW is similar to the alignment algorithm in FASTA [14]. However, with the hashing structure, SAP Mapper efficiently filter out most “seeding” sites before the application of the modified SW, which greatly accelerate the mapping process while ensuring the quality of the alignment. SAP Predictor uses a Bayesian model and a SNP filter for SNP-calling, and computes an alignment score for InDels-calling. Because of the typical memory consumption issue for hashing-based mapping tools, we benchmark SAP for short reads mapping using simulated and real exon sequencing data, and for long reads (∼1000 bp) mapping using simulated exon sequencing data and a real *E.coli* genome data sequenced using the third-generation of NGS technology. The benchmark results show that for short reads mapping, SAP mapper not only outperform MAQ, SOAP2 and Blat in detecting SNPs, but also achieve the fastest speed; for long reads mapping, SAP mapper is superior to BWA-SW and Blat in both the percentage of captured reads and the computational speed (a speed nearly 100× faster than Blat). In addition, SAP achieves high accuracy in detecting SNPs and InDels in the simulated data, and identifies more “valid” SNPs in the real exon sequencing dataset. As such, SAP offers the flexibility to deal with sequence reads generated by currently popular second-generation NGS technologies for polymorphism detection, while meeting the needs for mapping long sequence reads generated by the third-generation NGS technologies.

## Results

### Mapping reads to the reference

There are two mapping modes in SAP Mapper: FastMap and SlowMap that allow for exact hash look-up and inexact hash look-up, respectively, and are therefore evaluated separately in this study. Table 1 shows the time consumption of the compared methods, and Table 2 shows the proportion of captured reads by each method.

**Table 1:** Time-consumption by different algorithms in mapping reads from simulated data and real data (s).

Data type	Read length	Read coverage	SAP FastMap	SAP SlowMap	MAQ	SOAP2	Blat	SHRiMP
Simulated data	75 bp	5×	17.80	51.77	206.75	19.90	89.63	-
		10×	24.75	89.62	222.43	41.70	172.38	-
		20×	47.00	159.47	252.06	79.11	337.49	-
	150 bp	5×	20.93	33.97	184.47	22.08	179.50	-
		10×	33.73	51.80	186.24	46.61	303.91	-
		20×	56.39	100.43	193.43	81.39	679.51	-
	1000 bp	5×	22.07	28.27	127.63	31.17	1436.59	-
		10×	37.40	41.14	131.97	55.92	3326.57	-
		20×	57.95	70.61	148.68	84.05	5506.53	-
Real data	∼76 bp	∼40×	124.13	625.5	339.6	327.88	541.38	642609.1^a^

a : time-consumption was averaged across only seven individuals' exon-sequencing data.

**Table 2:** Percentage of captured reads by different algorithms.

Data type	Read length	Read coverage	SAP FastMap	SAP SlowMap	MAQ	SOAP2	Blat	SHRiMP
Simulated data	75 bp	5×	99.33	99.95	99.21	81.03	99.89	-
		10×	99.33	99.96	99.22	81.07	99.88	-
		20×	99.33	99.96	99.23	81.09	99.89	-
	150 bp	5×	99.87	99.99	0.00	45.92	100.00	-
		10×	99.86	99.99	0.00	45.94	100.00	-
		20×	99.86	99.99	0.00	45.92	100.00	-
	1000 bp	5×	99.24	99.53	0.00	3.18	99.08	-
		10×	99.20	99.49	0.00	3.13	99.04	-
		20×	99.23	99.52	0.00	3.18	99.07	-
Real data	∼76 bp	∼40×	92.05	93.10	92.79	88.33	92.54	95.00^a^

a : percentage was averaged across only seven individuals' exon-sequencing data.

For short reads mapping (< = 150 bp), overall SAP FastMap is the fastest among the five algorithms, followed by SOAP2, SAP SlowMap, MAQ, Blat and SHRiMP. In terms of the proportion of captured reads, in simulated data SAP SlowMap and Blat are comparable, both capturing above 99.5% reads. SAP FastMap captures slightly less number of reads than SAP SlowMap, while MAQ and Blat are significantly worse. Note that MAQ fails to capture any reads of 150 bp, while SOAP2's performance drops significantly when read length increases to 150 bp. In real data (about 54 bp), SHRiMP captures the most number of reads (95%), yet it is nearly 5000× slower than SAP. SAP SlowMap, Blat, MAQ, and SAP FastMap all capture above 92% of reads, with SAP SlowMap being the best of them (93.1%). SOAP2 is noticeably worse than the other methods, capturing only 88.3% reads.

For long reads mapping, in simulated data (1000 bp) SAP FastMap is the fastest method, followed by SOAP2, SAP SlowMap, MAQ, BWA-SW, and Blat. Depending on read coverage, SAP is about 20× faster than BWA-SW, and 60–100× faster than Blat. Note that the time-consumption of Blat is sensitive to read coverage. In terms of the proportion of captured reads, SAP SlowMap, FastMap, Blat and BWA-SW all capture more than 98.8% reads, with SAP SlowMap being the best one. For real *E. coli* genome sequencing data (about 900 bp), because they contain large number of errors, SAP FastMap is not evaluated. Blat is faster than SAP and BWA-SW, however this is because it does not find the exact match of most reads, and consequently only captures 2.98% reads. With default settings, BWA-SW captures only 17.5% reads with the time consumption of 30 m; with parameter tuning, it capture 61% reads with the time consumption of 250 m. In contrast, SAP SlowMap captures more than 62% reads by spending 15 m; with parameter tuning, it captures even 79% reads with the time consumption of 43 m. Therefore, SAP outperforms both BWA-SW and Blat for mapping long reads.

### SNPs detection

We use SAP Predictor to predict SNPs from the mapping results of SAP Fastmap and Slowmap, Blat, BWA-SW and SOAP2. SOAP2 has its own SNP prediction program, however for format problem it does not work well with its read mapping results. We employ MAQ's own SNP-caller to analyze its mapping result. SHRiMP is not evaluated, because it does not have a SNP Predictor and the format of its output does not fit SAP Predictor's requirement. Here, the mapping results of *E. coli* sequencing data are not analyzed, because the data contain high errors.

For simulated data, we know in advance where SNPs are located. Therefore, we can evaluate the accuracy and coverage of SNP calling. In general, the accuracy of SNP detection based on the mapping result of all methods except BWA-SW is above 97% irrespective read length and coverage (Table 3). This suggests that SAP predictor is able to accurately identify SNPs. The coverage of SNPs detection increases when read coverage or read length increases (Table 3). For example, based on the mapping results of SAP FastMap, the coverage of SNPs detection is increased from 53% to 94% when read coverage is increased from 5× to 20× and reads length is fixed at 75 bp, and is increased from 53% to 81% when read length is increased from 75 bp to 1000 bp and read coverage is set at 5×. Comparing the coverage of SNP detection in between the mapping results of different methods, we find that for short reads, SAP SlowMap corresponds to the best coverage, followed by SAP FastMap, Blat, SOAP2, and MAQ; for long reads, SAP SlowMap is also the best one, followed by SAP FastMap, Blat and BWA-SW. Thus, in the simulated data SAP is able to achieve the best performance on SNP detection using either short or long reads.

**Table 3:** Performance of different algorithms on SNPs detection in simulated data.

Read length	Read coverage	SAP FastMap		SAP SlowMap		MAQ		SOAP2		Blat	
		Cov (%)	Acc (%)	Cov (%)	Acc (%)	Cov (%)	Acc (%)	Cov (%)	Acc (%)	Cov (%)	Acc (%)
75 bp	5×	53.26	98.98	55.23	99.02	15.69	100.00	19.31	97.22	49.87	99.13
	10×	92.17	99.40	92.44	99.48	39.67	100.00	72.25	97.31	91.15	99.69
	20×	94.02	98.78	94.02	98.78	81.55	100.00	93.96	95.37	93.75	99.68
150 bp	5×	58.54	96.17	58.84	96.46	0.00	0.00	1.08	98.49	55.67	98.20
	10×	92.93	97.99	92.93	98.24	0.00	0.00	13.38	96.44	91.97	99.51
	20×	93.97	97.76	93.98	97.97	0.00	0.00	55.28	97.25	93.47	99.51
1000 bp	5×	80.70	95.25	81.13	97.96	0.00	0.00	0.00	0.00	76.09	98.98
	10×	94.23	97.11	94.28	98.33	0.00	0.00	0.00	0.00	92.12	99.79
	20×	95.43	98.60	95.43	98.87	0.00	0.00	0.00	0.00	92.90	99.86

Cov: prediction coverage; Acc: prediction accuracy.

For real data, the SNPs of each individual are not known. In order to evaluate a method's performance, here we consider a predicted SNP “valid” if it either has already been reported in dbSNP [15] or is predicted by at least two other methods (Table 4). Since SAP FastMap and SAP SlowMap are related, when one's results are evaluated, the other's results are not taken into consideration. On average, SAP SlowMap predicts 811 SNPs per individual of which 690 are “valid” (85.0%), while SAP FastMap predicts 773 SNPs per individual of which 679 are “valid” (87.9). In contrast, the number of valid SNPs by Blat is 568 out of 581 per individual, while this number is 590 out of 642 (91.9%) for SOAP2 and 596 out of 1050 (56.8%) for MAQ. Thus, in real data, SAP SlowMap is able to identify the most number of “valid” SNPs among the methods being compared.

**Table 4:** Performance of different algorithms on detecting SNPs in real data.

Individual number^a^	SAP FastMap		SAP SlowMap		MAQ		SOAP2		Blat	
	Stat^b^	Per (%)^c^	Stat^b^	Per (%)^c^	Stat^b^	Per (%)^c^	Stat^b^	Per (%)^c^	Stat^b^	Per (%)^c^
NA12878	577/659	87.56	700/815	85.89	510/897	56.86	515/554	92.96	502/515	97.48
NA18870	649/750	86.53	680/798	85.21	562/1217	46.18	563/624	90.22	542/559	96.96
NA18501	657/751	87.48	589/696	84.63	581/1107	52.48	581/633	91.79	534/551	96.92
NA15510	630/722	87.26	639/761	83.97	558/951	58.68	563/622	90.51	529/542	97.60
NA19240	754/840	89.76	670/791	84.70	665/1060	62.74	676/723	93.50	637/648	98.30
NA18507	760/871	87.27	773/924	83.66	671/1133	59.22	633/691	91.61	622/637	97.65
NA19137	654/727	89.96	796/922	86.33	571/1078	52.97	567/616	92.05	548/561	97.68
NA18861	711/815	87.24	693/803	86.30	645/1083	59.56	629/684	91.96	605/615	98.37
NA18956	666/747	89.16	686/790	86.84	574/982	58.45	555/601	92.35	552/566	97.53
NA12156	670/763	87.81	710/852	83.33	584/932	62.66	583/628	92.83	548/559	98.03
NA19129	710/812	87.44	662/793	83.48	617/1096	56.30	613/665	92.18	596/606	98.35
NA18856	676/757	89.3	667/771	86.51	606/1045	57.99	620/672	92.26	575/584	98.46
NA19143	611/741	82.46	721/845	85.33	511/1143	44.71	504/560	90.00	510/528	96.59
NA18517	779/868	89.75	661/758	87.20	682/1191	57.26	653/709	92.10	626/637	98.27
NA18555	687/777	88.42	629/789	79.72	608/981	61.98	586/627	93.46	595/604	98.51
NA10851	680/772	88.08	756/874	86.50	601/914	65.76	608/666	91.29	574/590	97.29
Average	679.4/773.3	87.87	689.5/811.4	84.98	596.6/1050.6	56.79	590.6/642.2	91.96	568.4/581.4	97.78

a : individual number was adapted from [16].

b : statistics, the left one is the number of “valid” SNPs, and the right one is the total number of predicted SNPs.

c : Percentage of “valid” SNPs in the predicted SNPs.

### InDel detection

The prediction of insertions and deletions is of great importance, for these variations may have a larger impact on gene functions. However, because all the other methods do not report InDels, here we only evaluate SAP's performance on detecting InDels in the simulated datasets (Table 5). SAP can detect InDels with high accuracy irrespective read length: the accuracy is above 97% and 87% for insertions and deletions, respectively, for reads with different length. The coverage of InDel detection is dependent on read coverage and read length, and in general increases as read length and coverage increase, highlighting the necessities of developing new NGS technology to produce longer sequence reads. For example, at 5× read coverage, the prediction coverage of deletions by SAP FastMap is improved from 12.3% to 54.7% when read length is increased from 75 bp to 1000 bp; for reads of 1000 bp, the prediction coverage of deletions is improved from 54.7% to 91.2% when read coverage is increased from 5× to 20×. Compared to SAP FastMap, SAP SlowMap can significantly improve the prediction coverage of InDel detection, especially when read coverage is low, indicating the usefulness of inexact hash-lookup for InDel detection.

**Table 5:** Performance of SAP on detecting InDels in simulated data.

Read length	Read coverage	Deletions				Insertions			
		SAP FastMap		SAP SlowMap		SAP FastMap		SAP SlowMap	
		Cov (%)	Acc (%)	Cov (%)	Acc (%)	Cov (%)	Acc (%)	Cov (%)	Acc (%)
75 bp	5×	12.38	98.04	21.02	98.77	5.41	100.00	13.19	95.15
	10×	50.41	99.09	69.06	98.91	44.57	87.09	60.75	87.12
	20×	83.34	99.83	86.49	99.66	83.41	89.63	86.11	89.11
150 bp	5×	29.62	97.09	37.94	96.01	21.84	94.82	28.74	95.16
	10×	81.20	99.40	85.24	98.50	67.06	93.13	72.67	90.90
	20×	86.14	99.14	87.19	98.81	82.04	91.36	82.73	91.29
1000 bp	5×	54.76	97.76	70.35	97.31	39.85	97.72	49.99	97.04
	10×	88.86	97.94	92.82	97.50	73.04	89.98	75.12	90.08
	20×	91.24	99.42	92.27	99.43	76.20	93.96	76.17	93.75

Cov: prediction coverage; Acc: prediction accuracy.

## Discussion

With the development of third-generation NGS, sequence reads are expected to become longer and contain more polymorphism information such as SNPs and InDels. For example, the average read length of the recent *E. coli* genome sequence data is about 904 bp with high error rate. This effectively rules out the use of most currently available reads mapping tools, as they often require very few number of mismatches in order to speed up the mapping process. Though Smith-Waterman algorithm is able to determine the exact displacement of long reads at the reference, it is very time consuming, making it computationally infeasible to apply SW directly for mapping and aligning long sequence reads. Therefore, there is a strong need for new alignment tool for efficiently mapping long reads containing high polymorphism information.

BWA-SW is a recently developed algorithm for long reads mapping, which uses the standard SW for alignment extension only if the “seed” sequence reads has a score above a certain threshold in the initial comparison. However, since a read is often mapped to different positions with positive scores, BWA-SW is still time consuming. In this study, we tackle this problem by introducing a modified version of SW that takes advantage of the hashing result to reduce its complexity to approximately *O(n)*. This has significantly speeds up the mapping process, making SAP the fastest method for reads mapping in the benchmarks. On the other hand, since SW is still used, the alignment is of high quality, which results in the high accuracy and coverage of SNP detection by SAP in simulated datasets. This makes SAP a useful tool to meet the needs of third generation NGS technologies for efficiently mapping long reads containing high polymorphisms information or with high error rate.

Although SAP is developed for long reads mapping, it is still well adapted for short reads mapping given that third generation sequencing technologies have not yet been widely applied. What makes SAP different from other “hash-lookup”-based algorithms are that: (1) SAP uses the “seeding” result to optimize the SW algorithm when aligning the read sequence to the reference genome, which greatly reduces the running time while guarantees that each read is mapped to the most possible location on the reference genome; (2) Unlike most other algorithms which require additional processing after alignment when determining SNPS and InDels, in SAP the information of SNPs and InDels are already contained in the alignment with the implementation of the SW algorithm. The above features of SAP make it an accurate and convenient algorithm for short reads mapping.

However, since hashing is used in SAP, it is expected that for large reference, such as the whole human genome sequence, memory consumption will be an issue for SAP, because it usually consumes a memory of about 30× larger than the size of the reference. This may be solved in the future by implementing the BWT techniques to index the genome sequence and apply the modified SW to align two FM-indices. Nevertheless, at current stage, SAP is well suited for mapping sequence reads to relatively small reference sequence, such as the human exome sequence or bacteria genome sequence.

## Materials and Methods

### SAP Mapper

#### Indexing and hash look-up

SAP uses a hash table to index sequence reads before the mapping process. By default, SAP cuts a read into seven pieces each with a length of 15 bp, which can be adjusted by user. For example, a read *R* (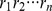, where n is the length of the read) is cut into seven pieces. The starting position of each piece is 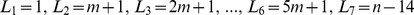, where 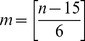, and the piece, *P_i_*, is 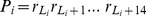. SAP Mapper searches with each piece, *P_i_*, against the reference sequence for a possible starting position of *R*, and define it as *l_i_*, where 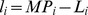, and *MP_i_* is the mapped position of *P_i_* on the reference. There are two mode parameters to control the hash look-up: the FastMap and the SlowMap mode that allow zero or only one mismatch in a given piece according to the reference, respectively. Finally, SAP will keep a read if at least two of the seven pieces are successfully mapped to the reference.

#### A modified version of the Smith-Waterman algorithm

After hash look-up, *l_i_* is sorted. Without the loss of generosity, we can suppose 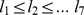. Then, SAP Mapper compares *l_1_* with *l_7_*. If 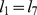, then the read does not contain any insertions or deletions, and a direct comparison between read and reference sequence is performed, with the score calculated as 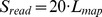, where *L_map_* refers to the number of identical nucleotides between the read and the reference. If 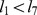, then it indicates the presence of InDels, and a modified version of the SW algorithm will be initiated to align the sequence read to the reference. A typical SW is to optimize function *F_i,,j_*, which is defined recursively as 
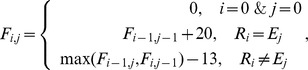
where *E_j_* is the 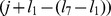-th position on reference sequences. After optimization of *F_i,j_*, the read is aligned to the reference by backtracking. However, the typical SW is very time-consuming, with a complexity of *O(nm)*, where n is the length of read, and m is the portion of reference that a read can be mapped onto. Here, we introduce a modified version of SW that takes advantage of the hash structure. After analyzing *F_i,,j_* during the calculation process, we find that in most cases, when 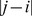 is very large, the value of *F_i,,j_* is not useful. Therefore, we require SAP Mapper calculate *F_i,,j_* only if 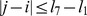. Since in most cases 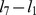 is quite small, the complexity of the modified SW is approximately *O(n)*, making it much more computationally efficient than the typical SW.

Finally, *F_i,,j_* is transformed into a score: 
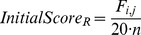
. Only when the 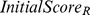 is greater than 0.9 would SAP Mapper keep the alignment and compute the final score: 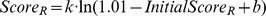, where 
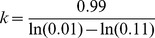
 and 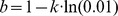. The final score 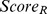 is used in predicting SNP and InDels.

### SAP Predictor

After reads mapping, SAP Predictor is used to compute the probability of a given site to be either SNP or InDels.

#### SNP prediction

At a given site in the reference genome, there are at most two different nucleotides. Therefore, to infer the genotype, we only need to consider the two most frequent nucleotides among the reads across that site. Suppose in an observation *D*, the two most frequent nucleotides are *b* and *b′*, with a frequency of *k* and *n-k* reads, respectively, where n is the total number of reads with either *b* or *b′*, then there are three possible genotypes (*Q)*: *<b,b>*, *<b,b′>*, or *<b′,b′>*. For genotype *<b,b′>*, its probability follows a binomial distribution, i.e., 
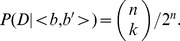
 This can be rewritten as 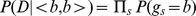, where 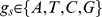 is the nucleotide observed in reference sequence. Here, 
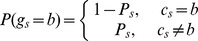
, where 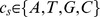 is the nucleotide on the read, and *P_s_* is the probability of sequencing error with 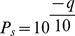, with q being the PHRED scores at the corresponding position of the read. The probability of genotype <b′,b′> is defined similarly. Similar to MAQ, we set a prior probability for the three genotypes as 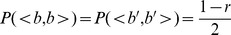 and 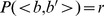, with *r* = 0.001. Finally, *P(Q|D)* is calculated using the Bayes Rule, with 
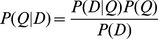
, where 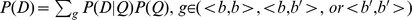. If the genotype with the highest probability is different from that on the reference, then it is tentatively called a SNP.

#### SNP filter

To filter out false positive of SNP calls, we develop two SNP filters: a read depth filter, and a score filter. The read depth filter checks the depth of reads at a given site, and by default is set at *Cover/5*, where *Cover* is the average coverage of the sequencing data, and is computed as 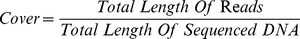. The score filter checks the quality of a predicted SNP, with a lower score indicating a better reliability in our model. The score is calculated as 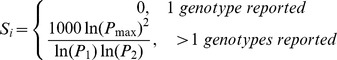, where *P_max_* is *P(Q|D)* with the highest probability, and *P_1_* and *P_2_* are the other two probabilities.

#### InDel prediction

SW can automatically detect InDels when aligning the reads. Suppose an insertion is detected on a read, the quality score of the insertion is calculated as 
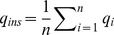
, where *q_i_* is the quality of *i*-th site on the read. Then, SAP Predictor sums all *q_ins_* in that position and compares it with the quality data of the nearby sites. If *q_ins_* is greater, an insertion would be reported. The prediction of deletions is done in a similar way. SAP is publically available at https://github.com/davidsun/SAP.

### Benchmark SAP

SAP Mapper and SAP Predictor are evaluated separately. The following benchmark datasets are prepared: the simulated human exon sequencing data with both short and long reads, the real human exon sequencing data with short reads, and the real *E. coli* genome sequence data with long reads.

#### Simulated exon sequencing dataset

In simulation, we first randomly introduce SNPs and InDels into the human genomic sequence (hg18 assembly), with a probability of 0.02% and 0.009%, respectively. Then, we simulate the exon-capture sequencing process by cutting each exon randomly into small DNA fragments with length of 300–1500 bps. The number of DNA fragments generated from each exon follows a Poisson distribution. Finally, with a given read length, e.g., 75 bp, we generate the single-end reads from either the 3′- or 5′-end of the fragments with an error rate of 2%. By controlling the number of fragment-cutting and read-generating, we obtain sequencing data with different read coverage. The advantages of using the simulated data are that (1) we know in advance where the SNP and InDels are located in the reference, and (2) we can control the sequencing error, read length, and read coverage, making it possible to evaluate the performance of the program and make appropriate improvements in a dynamic way. Here, for each combination of read coverage (5×, 10×, and 20×) and read length (75 bp, 150 bp, and 1000 bp), we generate three datasets following the above procedures.

#### Real exon sequencing dataset

We download the exon-capture sequencing data of 16 individuals from a recently published article [16]. There are approximately 8.4 million quality-filtered reads with a length of 76 bp (including a 22 bp primer) for each individual's exome. The read coverage is over 40×.

#### Real E. coli genome sequence data

We download a recently sequenced *E. coli* O104:H4 genome data using the third generation NGS technologies. The average read length is about 902 bp. The read coverage is about 75.

#### Other alignment tools

We compare SAP with five alignment tools: BLAT, MAQ, SOAP2, SHRiMP, BWA-SW. BLAT was downloaded from http://users.soe.ucsc.edu/~kent/src/. MAQ was downloaded from http://maq.sourceforge.net. SOAP2 was downloaded from http://soap.genomics.org.cn. SHRiMP was downloaded from http://compbio.cs.toronto.edu/shrimp. BWA-SW was downloaded from http://sourceforge.net/projects/bio-bwa/. All alignment tools are tested with their default settings. For the real *E. coli* data, the BWA-SW setting of (-b5 -q2 -r1 -z10) is also tested following the recommendation in its documentation.

#### Evaluation measures

All methods described above are evaluated on an IBM computer server with Intel(R) Xeon(R) E5405 CPU of 2.00 GHz with 16GB memory installed. One CPU thread is used. For the simulated dataset, the prediction accuracy of SNP or InDel detection is defined as TP/P, where TP is the number of true SNPs or InDels predicted by a program, and P is the total number of predicted SNPs or InDels. The prediction coverage is defined as TP/T, where T is the total number of true SNPs or InDels. For each combination of read coverage and read length, the prediction accuracy and coverage are averaged across the three datasets.
